# Medico-Chirurgical Transactions

**Published:** 1816-07

**Authors:** 


### *Medico-Chirurgical Transactions, Vol. vi.*

( Continued from Vol. i. p. 485. )

### *A new Method of tying the Arteries in Aneurism, Amputation, and other Surgical Operations, &c.*

By William Lawrence, Esq.

Dr. Jones's Treatise on this subject, banished thick ligatures, tapes, cylinders of cork, linen compresses, and various other clumsy contrivances, from the practice of English surgeons; though it is pretty evident, that such is not the case among our continental brethren, who are very slow in availing themselves of the improvements introduced by the "haughty Islanders." The use of the ligature, under the modifications suggested by our present knowledge, may be considered as arrived at perfection. Still, ligatures, as foreign bodies, must cause irritation and suppuration; and where, as in amputation, they are multiplied, the union of the wound is retarded, while the pain and spasm are sometimes considerable. Are these evils inseparable from the use of ligatures? Or is there any plan by which we can avoid them? Mr. Lawrence thinks there is, and has adopted the method of "tying the vessels with fine silk ligatures, and cutting off the ends as close to the knot as is consistent with its security." The foreign body is thus reduced to the very insignificant quantity which forms the noose round the vessel, and the knot itself. The silk necessary to secure a large artery in this manner, weighs from $\frac{1}{50}$ to $\frac{1}{60}$th part of a grain. These ligatures do not interfere with the process of adhesion, and we need hardly entertain any serious apprehension, that substances so minute will excite subsequent irritation.

"That kind of silk twist, which is commonly known in the shops by the name of Dentist's silk, and which is used in making fishing lines, is the strongest material I know of, in proportion to its size, and therefore the best calculated for our purpose, which requires considerable force in drawing the thread tight enough to divide the fibrous and internal coats of the arteries." p. 164.

In experiments with large and small ligatures, Mr. Lawrence found that the *former* completely divided the internal and fibrous tunics of the vessels; *detached* them extensively from the external, and left a very thin stratum of the cellular coat uncut. The strongest *twist* had a similar effect; but the remaining stratum was not so thin, nor were the divided tunics so much detached. The weaker silk ligatures did not in general, completely cut through the two internal coats; sometimes the fibrous tunic was divided, the internal remaining nearly entire; or some fibres of the middle coat were uncut; or shreds of the fibrous and internal were left. Sometimes there was a simple, clean cut of these tunics, not going completely through: there was no detachment of the coats, and a considerably thicker external portion remained undivided.

"That the effects of the small ligature, as detailed in the foregoing trials, are much the best calculated to promote healthy adhesion of the arterial coats, and to maintain them in contact, until this union has become firm enough to resist the impetus of the blood; while the thick cord is most likely to produce sloughing of the detached external stratum, to be discharged before the end of the vessel has united firmly enough to withstand the force of the blood, and thus to expose the patient to the risk of secondary hæmorrhage, are points too obvious to require further illustration. Hence, if any proportion is to be observed between the size of the artery, and of the ligature, it should be an inverse one: the large vessel requiring a small ligature, while a small artery may be tied without danger with a large one.

Mr. Lawrence here anticipates a question that may be put, namely, What becomes of these ligatures? From experiments on brutes, he apprehends that, "the layer of lymph, which is so quickly effused on cut surfaces, covers and incloses these small threads, which must therefore remain in the wound." p. 170. In our review of Mr. Guthrie's work, we stated that gentleman's remarks on this subject. In two or three instances he saw ill looking abscesses formed by the retained ligatures. As Mr. Lawrence observes, this circumstance may have been owing to the size of the thread used. Mr. Lawrence himself has not met with any such occurrence, though he has employed the method in question in ten or eleven cases of amputation; in six operations on the breast, and in the removal of two testicles. This plan, at all events, supersedes the necessity of threads hanging out of the wound, the management of which is often so troublesome and painful. The following are Mr. Lawrence's excellent observations on amputation.

"Cutting the ligature short removes from the wound the principal obstacle to its speedy union: the same object must be kept in view in other parts of our treatment. When the process of adhesion fails, it is from the inflammatory process running too high, and passing into the suppurative stage. We should, therefore, attempt to diminish the action of the vessels, which is most effectually accomplished by reducing the temperature of the part. Is this object likely to be gained by covering the whole surface with adhesive straps, by tight bandaging, by flannel rollers, tow pledgets, and worsted caps. When the vital powers of a limb are impaired by the interruption of its arterial trunk, in the operation of aneurism, we envelop the part in flannel, and use other means to prevent the dissipation of its heat. The opposite treatment is required after amputation. The parts are to be gently brought in contact by strips of adhesive plaster, avoiding all force and strain, which are very painful and injurious, when the subsequent tumefaction comes on. Instead of carefully covering every part of the edges, I rather leave intervals between the straps, at which the blood may escape, if there should be slight oozing; with this view, it is well to leave the inferior angle of the wound open. A small bit of lint spread with white cerate should be applied on the parts which are not covered by the adhesive plaster. A soft folded rag dipped in cold water, or in a saturnine solution, and squeezed out, should then be laid over the stump, and be kept constantly damp; the limb itself being covered by a sheet only. It is hardly necessary to mention, that under some circumstances these precautions against too great action are not required; that the rags are to be used damp, but not dripping wet, so as to inundate the bed, and give the patient cold; and that it would be contrary to principle to chill the parts. Such appears to me the surest method of promoting adhesion, of keeping down inflammatory action, and thereby preventing spasms, secondary hæmorrhage, and protrusion of the bone."

Mr. Lawrence here introduces several very interesting cases, accompanied by equally interesting and judicious remarks. In a most unfavourable necrosis of the femur, the bone was sawn through obliquely in the trochanter major, so as to remove the exterior and lower half of that process.

"The head of the bone," says he, "might have been very easily removed from the socket in this case: perhaps the proceeding I have described would be found an eligible mode of amputating at the hip joint; the bone being first sawn through at the trochanter major, after a *circular* incision of the soft parts, and the remainder subsequently removed from the stump."

We do not see any use in first sawing the bone and then removing the remaining portion: why not remove the head of the bone from the socket after it is stript by the circular incision, using the bone itself as a lever to facilitate its disengagement from the acetabulum. Such a mode of amputating at the hip joint was proposed some months ago in the New Medical and Physical Journal, vol. 8, p. 517. Where the plan proposed was almost verbatim what Mr. Lawrence here states.

In a discussion relative to the propriety of *immediate* amputation in cases of injury requiring that operation, Mr. Lawrence takes the part of Mons. Larrey and our able countryman Mr. Guthrie. In the naval service, where the nature of the injuries (cannon shot wounds) has always admitted of less hesitation than in military or civil life, the question has long ago been decided. Can any one doubt, that in the great sea fights which immortalized Howe, St. Vincent, Duncan, and Nelson, the almost innumerable amputations which took place immediately after each action *with success*, were not sufficient memorials and proofs of the utility and safety of primary amputations. Yet we now find the question agitated as though the foregoing records were buried in oblivion, or had never existed!

Mr. Lawrence also inclines to the opinion, now spreading in the medical world, that amputation may sometimes be safely had recourse to, where gangrene is *spreading* along a limb, without waiting for a cessation of the morbid process, or separation of the unsound parts. In confirmation of this opinion, he relates the case of a man on whom he performed amputation at the shoulder joint, while mortification was rapidly advancing up the arm. Mr. Lawrence judiciously observes, that he does not mean to recommend this practice in all cases of mortification from local injury, since gangrene arising in an unsound constitution from a comparatively slight accident, may be regarded as the result of constitutional disposition, rather than of the local cause. Under such circumstances amputation would of course be hopeless. It is only in mortification following very severe injury, in a subject otherwise healthy, that the plan in question is at all applicable.

Several interesting cases are next related, which our limits prevent us noticing; we shall select, however, the following advice respecting the operation for aneurism.

"After dissecting down to the artery, a slight scratch or incision may be made, through the sheath, close to the side of the vessel. Then, with a narrow aneurism needle, nearly pointed at the end, and made as thin at its edge as it can be without cutting, a single silk ligature is to be conveyed round it, the point of the needle being kept in contact with the artery. A needle of this form makes its way easily through the cellular substance, and the vessel is detached only in the track of the instrument. I should recommend a middle sized washed silk ligature, which should be drawn moderately tight; under which circumstances the thread may probably remain permanently on the vessel; at all events, ulceration or sloughing of the external coat will not occur so soon, as when greater force is used in drawing the knot. The ends of the ligature being cut off near the knot, the wound is to be united as a simple incision."

This article is very creditable to the well known talents of its author.

*Two Cases of the true Elephantiasis, or Lepra Arabum.*
By William Lawrence, Esq. and Dr. Southey.

The first Case was a lad, born in America, but resident the greater part of his life in one of the Bahama Islands. He was of English parentage, and seems to have contracted the disease on his passage to England in 1813. No medicine was of any service, and Nature at length effected a cure of the external disease, though symptoms of pulmonary consumption succeeded. There is nothing interesting in the other case—it remains uncured.

### *On some Affections of the Larynx requiring the Operation of Bronchotomy, (Tracheotomy, or Laryngotomy).* By William Lawrence, Esq.

The first Case was a man, about 45 years of age, who during a mercurial salivation took cold, became hoarse, and affected with considerable dyspnœa. His breathing was accompanied with a peculiar sound, and could only be performed when sitting upright.

"His opened mouth, elevated shoulders, and spasmodic struggle of the whole frame, marked the narrowness of the passage for the air; while the cold and pallid skin, the small, feeble, and rapid pulse, indicated clearly enough the general debilitating effects of such imperfect respiration."

In about four days from the attack, the case was regarded as desperate by the physician, and Mr. Lawrence performed tracheotomy, which gave relief for a time, but the patient sunk in eight days. The chordæ vocales, sacculi laryngis, epiglottis, &c. were perfectly healthy, as well as the lining of the trachea. The rima glottidis of its natural dimensions. A small portion of the cricoid cartilage was ossified and loose. The chest exhibited extensive marks of recent disease; inflammation, and effusion. The obstruction to respiration in this case, was most probably owing to *spasmodic* stricture of the glottis.

Case 2.—A woman, 35 years of age, was admitted into Bartholomew's with great dyspnœa. She was obliged to sit upright; with her mouth open; eyes protruded; great agitation, and quick breathing. No local pain; no dysphagia. Pale and cold skin; rapid and feeble pulse. Tracheotomy was performed, and the patient breathed through the opening with complete relief. The next day, however, the breathing was impeded, and an attempt to remove a portion of the trachea failed. She died the next day. The membrane of the chordæ vocales, sacculi laryngis, and front of the arytenoid cartilages possessed its natural pale colour, but was thickened and granulated on the surface so as to completely shut the rima glottidis. The affection, confined entirely to the parts enumerated, occupied a very inconsiderable extent of the membrane. The trachea, epiglottis, and neighbouring parts, and the thoracic and abdominal contents were perfectly sound.

Mr. Lawrence brings forward from various sources, foreign and domestic, a number of cases analogous to the preceding, from which the following conclusions are deduced.

"1. That the larynx is subject to affections differing considerably in the nature of their symptoms, and in their progress; but resembling each other in their ultimate effect, of obstructing the passage by which air is received into the chest.

"2. That the difficulty of breathing amounting to a sense of suffocation, the sound produced by the passage of the air, the affection of the voice, which is either extremely hoarse or reduced to a scarcely audible whisper, in many cases pain of the throat, and difficulty of swallowing, together with the absence of symptoms indicating affection of any other organs, are the signs by which this obstruction may be recognized.

"3. That the impeded state of respiration causes a violent constitutional disturbance in the acute cynanche laryngea, while it has a general debilitating influence in the more chronic forms of the disorder; and that these effects are in themselves fatal, after a certain time, even if the original obstruction be obviated.

"4. That local and general bleeding, blisters, and the various internal means, are usually inefficacious.

"5. That the operation of bronchotomy, by providing an artificial opening for the air, produces complete relief; but, for the reasons mentioned under the third head, it is ineffectual, unless performed very early.

"6. That the operation is free from danger, has been many times successfully performed, and has not in any instance produced unpleasant consequences.

"The operation itself is so simple and easy, that there is very little to be said on the mode of executing it. Of the three situations in which it has been proposed to make the opening, viz. in the thyroid cartilage, between that and the cricoid, or in the trachea, I consider the first as least eligible. Besides the objections from the ossification of the cartilage, and the danger of wounding or otherwise injuring the chordæ vocales, there is the inconvenience in the case of angina laryngea, arising from the swollen and thickened state of the membrane, which may actually impede the passage of the air. I am not aware of any objection to a transverse opening between the thyroid and cricoid cartilages. The prominence of the former in the neck, serves as a guide to the part which should be opened. The only sources of embarrassment in the two cases I have related, were the very considerable and rapid motions of the larynx accompanying the obstructed respiration, and the necessity which the patients felt from the same cause, of keeping the head erect. When the air tube has been opened in this part, a short flattened canula, curved so as to correspond with the axis of the trachea, should be introduced. In some instances, this has been borne quietly in the trachea, while in others it has produced so much irritation, cough and sense of choking, as to render its immediate removal necessary.

"Under the latter circumstances, I should advise a longitudinal incision, of about half an inch, in the middle of the trachea, and the removal of a thin slip of the tube, which will leave an artificial opening for respiration, equal in size to the natural one.

"If there should be troublesome hæmorrhage from any branch of the thyroid artery, or from a thyroid vein, the bleeding vessel ought to be secured before the trachea is opened."

*Cases subsequently to the reading of the foregoing Paper.*

David Jones, 53 years of age, became affected, on Friday, the 24th of June, with hoarseness and slight pain in the throat. Took some opening medicines. On Friday, the 7th of July, experienced difficulty of breathing; which was performed with a *whooping noise.* Voice reduced to a whisper. Bled and purged on the 8th. 12th. Became suddenly much worse; dyspnœa great; constant jactitation. An emetic gave some relief; throat covered with a blister. The difficulty of breathing still increasing, he was sent to Bartholomew's Hospital. Here the dyspnœa was excessive; sweat poured down in streams from the whole body; the pulse was 120, full, strong, and intermittent. No dysphagia; no inconvenience from fully distending the chest. Bronchotomy was determined on.

"I made a perpendicular incision, cut through the cricoid cartilage, and neighbouring part of the trachea, and removed a sufficient portion of these parts to leave a free opening for respiration. The blistered state of the skin, the depth of the parts in a short thick neck, the rapid motions of the larynx, and the entrance of blood into the tube from the vessels divided in exposing it, produced greater difficulties in the operation than might be expected." p. 252.

He breathed freely through the artificial opening; all the agitation and distress ceased; the skin became cool; the pulse soft; sleep ensued. The pulse, however, was rapid and intermiting for a few days, but he was free from fever. Breathing was entirely performed thro' the wound, and the voice lost. On the 21st. he was able to sit up. On the 5th of August, the breathing was performed entirely through the natural passage, and the wound was cicatrized on the 10th, when he was discharged cured.

The other case happened at the Middlesex Hospital, under Dr. P. Latham. Laryngotomy was performed by Mr. Charles Bell, and the patient was instantaneously relieved; but great difficulty was experienced in keeping the wound clear of the mucus and pus secreted there and in the neighbourhood. She lived from the 2d of August till the 11th of September, breathing entirely through the artificial opening, which consisted "in a division of the cricoid cartilage in front." She expired in a paroxysm, and dissection shewed the following morbid structure of the larynx.

"The mucous membrane, where it lines the larynx, and extends over half the posterior surface of the epiglottis above; and to about an inch beyond the cricoid cartilage below, had assumed, for the most part, a thick and puckered condition, and had partially thrown out coagulable lymph of a stringy and fimbriated texture, which obliterated the ventricle of the larynx, and contributed almost to close the rima glottidis. There were found besides, two distinct ulcerations through the substance of the thyroid cartilage, which contained pus, and communicated with the cavity of the larynx." The lungs, trachea, &c. were sound.

Laryngeal diseases present themselves so frequently, both in the chronic and acute states, and are, many of them, so extremely intractable in their nature, and terrible in their symptoms and consequences, that we must often see our patients die, unless the operation of tracheotomy is performed. Hence it is very desirable to know, under what circumstances the operation would be attended with a probability of success; and this question can only be solved by the multiplied experience, and the faithful detail of cases. The larynx is of so complicated a structure, that the exact nature of its diseases can seldom be ascertained but by dissection. To vindicate the propriety of the operation therefore, it is sufficient to know, that the disease is situated in the larynx, and that the patient's life is threatened.

"The distinction of the cause, as Dr. Latham justly observes, will be lost in the magnitude of the irritation; so that whether there be inflammation of the membrane, or ulceration of the cartilages, abscess in the cellular substance, or accidental lodgement of an extraneous body, the symptoms will probably be the same."

The next article in this volume is a very interesting train of experiments on the formation of bone, by Mr. Howship, a gentleman whose professional ardour and natural abilities, are making him well known in the medical world. It would be useless to detail the experiments without the figures, to which constant references are made: but we shall present our readers with the conclusions drawn by our ingenious author in his own terse and descriptive language.

"1. That in the mammalia, the first rudiments of ossification in the long bones are the effect of a secreting power in the arteries, upon the internal surface of the periosteum, which produce a portion of a hollow cylinder; this form of bone having been found antecedent to the evolution of any cartilaginous structure.

"2. That at a certain stage of the process, the mode of operating is changed, in order that it may proceed more expeditiously. A cartilage is formed, which, by the nature of its organization, and by admitting of a specific provision of cavities and canals lined with vascular membranes, which secrete an abundant store of gelatinous matter, is adapted to this particular purpose; while at the same time, it serves to determine the future figure of the extremity of the bone, by establishing and conducting the ossifications within its own substance.

"3. That from the appearance and texture of cartilage, when examined under the microscope, it may be defined, an even and finely granulated albuminous matter, deposited in the interstitial spaces of an exceedingly elastic bed of semi-transparent reticulated structure, which is apparently a modification of gelatine.

"4. That from the period when the ossification proceeds in the mode above described, by the medium of cartilage, the process is continued in the same uniform manner till it has completed the growth of the bone. The growth of the epiphyses, and their union with the ends of the bone, are also effected by the same means.

"5. That the ossific matter in the cylindrical bones is deposited primarily in the form of fine thin tubular plates: a mode of deposition of all others the most favourable for their being subsequently remodelled, and for facilitating all the subsequent changes of structure they are destined to undergo.

"6. That while the circulation in the capillary arteries situated between the cartilage and bone must provide the phosphate of lime; the principal agent in extending the cylinder, and in effecting the subsequent progressive changes of structure, which in a growing bone are continually taking place, appears to be simply the mechanical pressure exerted by the fluid secretions within the medullary cavities of bone, this power operating successively in different directions, according to the particular determination given by the circulation.

"7. That the mode of circulation most favourable for ossific action, is a very slow and uniform motion of the blood through the capillary system; and that the numerous inflexions of the minute arteries in the pericranium, and the great weakness, and rectangular mode of giving off the smaller arteries upon the dura mater, as well as the extremely curious appearance of the blood and injected matter, upon the fine membranous linings of the canals in cartilage, indicating, as I believe, something beyond a mere capillary circulation, are to be considered as so many evident provisions for securing this condition.

"8. That in the formation of the cylindrical bones, the ossific surface is arranged into tubular plates of two different sizes, constituting a larger and a smaller series; an arrangement by no means essential to the increase of a bone, because in many of the early stages of ossification, and also where the growth is very slow, the larger series is found to be entirely wanting.

"9. That the only apparent use of the larger series of tubes, is that of augmenting the quantity of blood circulated through the ossifying structure, so as to increase the rapidity of growth; for they are abundant in animals of quick growth, less numerous in those that reach maturity slowly; and in the same animal I have observed they are employed by nature, or laid aside, in conformity with the quick or slow developement of structure, which we know actually takes place at the particular period when the examination is made.

"10. That in the growth of the cylindrical bone, and of those flat bones that are formed upon cartilage, the deposit of the ossific secretion is in the first instance made around the external openings of the smaller series of tubes, and upon these only. This opinion derives support from the recent appearances of the bones of quadrupeds, but is most clearly established by the characters found upon the ossific surface in the bones of birds, where the gradations of progressive evolution are more readily traced.

"11. That in the flat bones of the skull, the circumstances under which ossification takes place, differ materially from those above described. In these the phosphate of lime, in combination with the animal mucilage, is occasionally deposited in small detached unequal masses, without regularity, as if merely laid in the way, preparatory to their subsequent application; that these soon become connected with the more central parts of the bone, and are found to decrease in thickness, as they increase in breadth, until they are finally consolidated with the original plate of bone.

"12. That the particular simplicity observable in the mode of production of the bones of the skull, affords a strong argument in favour of the opinion, that pressure variously modified, constitutes one of the most efficient instruments in the hand of Nature: for in this instance, the uniform, though gentle pressure from the impulse of the circulation, and the constantly increasing volume of contents in the head, must be admitted to be the sole agents in completing that process, which in its commencement had the appearance of being conducted in a comparatively imperfect manner.

"13. That the ultimate texture of bones is not laminated, but reticulated, the phosphate of lime being deposited in an interstitial substance; for although from the greater compactness necessary to the bones of quadrupeds, the ultimate structure is not in them so readily traced, yet in the more delicately constructed bones of birds, this mode of arrangement is sufficiently obvious, and may at any time be readily ascertained."

*Observations on the Mediterranean Fever.* By ALEXANDER DENMARK, M. D. Physician to the Fleet in the Mediterranean.

THE present document adds one more confirmation to the statements of late writers respecting the utility of venæsection, purgatives and mercurials in fevers of the warmer latitudes. In the Mediterranean fever Dr. Denmark observes, venæsection was not performed till reaction had commenced after the cold stage of the paroxysm; a circumstance that should always be borne in mind. He did not find much difference in the effect, whether the blood was taken from the temporal artery, or the brachial veins, though a prominent feature of the disease was determination to the head. He acknowledges, that abstraction of blood was not an infallible means of arresting the progress of the fever; though "This circumstance can by no means shake the general rule, supported as it is, by the best criterion of its utility—unparalleled success." p. 305. The danger, says Dr. D. consists in either applying the remedy too late, or too often. He did not, in general, find venæsection borne with impunity, after the yellow suffusion on the skin. Dr. Denmark speaks in high terms of mercury in the Mediterranean fever, and we offer the following passage for food to the anti-mercurial croakers, so numerous among the old women of the faculty.

"Viewed in any way, the utility of mercury is incontrovertible. Calomel is the preparation usually had recourse to; and it is undoubtedly beneficial in whatever way it operates. Whether it produce catharsis, when exhibited with a view to salivate; *or salivate when intended to act as a cathartic*, the result, in either case, will be beneficial." p. 306.

During the Summer of 1810, Dr. Denmark superintended the medical department of the navy in the Mediterranean. Some of the ships were then visited, to an alarming extent, with the endemic of the climate. The fever was, as usual, confined to the ships whose crews were laboriously employed in wooding and watering in harbour; exposed to an intense and reflected heat during the day, and to the chilling dews of the night, with the frequent abuse of spirituous and vinous drinks.

Dr. D's practice at this time was, to exhibit calomel and antimonial powder, three grains of each, every three or four hours, which combination seldom failed to procure copious bilious stools, and relief of the symptoms. Subsequently, however, Dr. D. gave calomel in scruple doses from the first invasion of the disease. In plethoric habits, this plan only produced copious alvine evacuations during the first days.

"But after the lapse of two or three days, and the use of free venæsection and purging; and at an earlier period in debilitated subjects, and in cases of relapse, the mouth often became suddenly sore with profuse ptyalism; and rapid convalescence as certainly ensued. I do not recollect any deaths after the specific action of the mercury shewed itself." p. 308.

This is a very strong confirmation of the plan pursued by Mr. Johnson seven years before, in a distant quarter of the world, and with similar success. Vide "*Tropical Climates, passim.* Dr. Denmark also employed purgatives, particularly mercurial ones; the cold affusion; the tepid bath; blisters, and in some cases, with caution, antimonials.

Dr. Denmark is among those who consider the bilious remittent of the Mediterranean as a *grade* of the concentrated or yellow fever of the West Indies, and makes no allusion to what has been termed the Bulam fever. In respect to the seat of the disease, he does not coincide with Dr. Clutterbuck.

"For I cannot allow," says he, "that in any of the fevers now under consideration, although the brain, stomach, intestines, liver, or all the viscera of the body be found deranged in structure, these derangements had any thing to do with the production of the fever. These morbid changes are *consequent* on the fever, and they are moderated or prevented by subduing the violence of the reaction." p. 316.

In this opinion we fully coincide; and we apprehend, that the theory of the ingenious physician above alluded to, is less strenuously maintained than heretofore.

(To be continued.)

